# Molecular detection and analysis of beak and feather disease viruses in Iran

**DOI:** 10.3389/fvets.2022.1053886

**Published:** 2022-12-01

**Authors:** Sara Dolatyabi, Seyed Mostafa Peighambari, Jamshid Razmyar

**Affiliations:** Department of Avian Diseases, Faculty of Veterinary Medicine, University of Tehran, Tehran, Iran

**Keywords:** BFDV, circovirus, Psittaciformes, phylogenetic analysis, molecular analysis, *rep gene*, *cap gene*, Iran

## Abstract

The beak and feather disease virus (BFDV) is one of the few pathogens capable of causing extinction of psittacines. To determine the prevalence and the nature of BFDV mutation, this study investigated the presence of the BFDV among 1,095 individual birds of the 17 psittacine species in Iran followed by analyzing the DNA sequences of seven replication-associated protein (rep) and 10 capsid (cap) genomes of the virus. The BFDV was found to be the foremost pathogen among more than 12 psittacine species, and phylogenetic analysis showed that the BFDV GenBank-published sequences from Poland, Saudi Arabia, South Africa, Taiwan, and Thailand were most similar to those of this study. Evolutionary analysis concluded that arginine, leucine, and glycine were the amino acids frequently involved in the least-conserved substitution patterns of BFDV, and conversely, methionine, glutamine, and tryptophan were the amino acids that exhibited ultra-high conservation through the substitution patterns. The high substitution rate of arginine to lysine and glycine to serine also made greater contribution to the BFDV gene mutation. The relative synonymous codon usage between two genes revealed that the cap genome encoded proteins frequently used fewer codons, while the rep genome encoded proteins used more codons only at moderate frequency, explaining the broader divergence of the cap compared to the rep sequence. The data analysis also introduced a new variant of BFDV that exists in the rep and cap sequences of budgerigars. While the existence of more new variants was suspected, more solid evidence is required to substantiate this suspicion.

## Introduction

The beak and feather disease virus (BFDV) that belongs to the genus Circovirus of the *Circoviridae* family (1) is the most significant wildlife pathogen causing a dermatological condition that threatens a wide variety of Psittaciformes (2–4). Psittacine circovirus, originally identified as the virus causing psittacine beak and feather disease (PBFD), was subsequently renamed beak and feather disease virus (BFDV) to reflect its causative agent. This disease is now considered to have become distributed worldwide largely due to international legal or illegal trade in exotic Psittaciformes (5, 6). Since BFDV has high environmental persistence and is capable of switching among closely-related hosts, it has spread rapidly throughout the world (5, 7).

The clinical signs of BFDV infection can range from a mild subclinical condition without symptoms to a severe clinical condition characterized by feather dystrophy and abnormal beak and claw development (1, 8, 9). While infected birds are often asymptomatic, they play an important role in spreading the virus (3, 10–13). Clinical signs are not limited to feathers but can also be found in the upper and lower beak of the affected birds as discoloration, progressive elongation, and palatine necrosis (14–16).

The prevalence of BFDV in Psittaciformes has been the subject of investigation by many researchers (17–22). Bassami et al. (17) reported eight BFDV isolates in Australia with nucleotide sequences ranging from 1992 to 2018 bp in size and an overall nucleotide similarity of 84 to 97% compared to previous reported Australian BFDVs. In the ensuing years, Ritchie et al. (18) reported BFDV infection occurring in 21 out of 25 different species of parrots in New Zealand whose testing revealed three distinct branches, each associated with a particular group of parrots. Within an endemic population, De kloet et al. (19) detected six clusters along with five new BFDV strains. A study by Julian et al. (21) also indicated that BFDV can be mutated within a captive population by introducing new variants originating from captive parrots. Similarly, Ma et al. (22) revealed three new distinct BFDV strains within budgerigar species in China.

While BFDV sequence has seven open reading frames (ORFs), three frames in the viral string and four frames in two strings, in a sequence that complements the repetitive sequence (8, 23, 24), among those ORFs only two, ORF C1 and ORF V1, are significantly influenced by structural and genetic changes. ORF V1 is located on a viral strand and is thought to encode replication-associated protein (Rep), a protein involved in rolling circle propagation (RCR) (24, 25). ORF C1 is located in the complementary strand encoding a structural component of the viral capsid (*cap*) gene (8, 24). The functional domain of the *rep* gene usually remains intact and activates RCR to form the ssDNA sequence, thereby initiating cloning sequences by binding to the host cell (25–27). A hypothetical stem-loop structure with a conserved nanonucleotide sequence motif (TAGTATTAC) has been found in the intergenic region of the sequence, where circoviruses bind to their host DNA polymerase (28).

It is widely agreed that the BFDV is prone to mutations that enable the circovirus to become a host generalist and retain virulence by facilitating flexible host-switching (4, 27, 29–31). A high BFDV mutation rate was initially postulated by a study conducted by Raue et al. (32), a study in which many parrot species were infected by BFDV, with further analysis showing that changes in the specific secondary sequences of the *cap* gene with structural changes may contribute to the spread of BFDV and weakened immunity in infected birds. In the ensuing years, Raidal et al. (4) claimed that the capsid gene is evolving faster than the gene encoding Rep protein, reflecting high potential for sequence divergence and contributing to the belief that BFDV exhibits a high rate of mutation due to structural limitations of encapsidation (4).

Based on a literature review, BFDV has a high potential for genetic evolution, depending both on the host condition and geographical characteristics. It is well-known that BFDV has a complex morphology, but the functional genomic basis of BFDV is poorly understood and current knowledge is restricted to a small number of studies investigating virus morphology. Also, with respect to the high capability of BFDV evolution due to geographical characteristics of the host, more epidemiological investigation is required in Iran. This study was therefore conducted to first evaluate the current prevalence of BFDV within Psittaciformes in Iran and then detect any possible evolution occurring within the Psittaciformes population of our country. This study was ultimately aimed at investigating the mutation genomic basis of the BFDVs in Iran.

## Materials and methods

### Samples

Plucked contour feather samples were collected from 1,095 individuals of 17 psittacine species over an interval of two consecutive years (January 2018 - December 2019). Three to five feather samples for each individual bird were submitted to the molecular diagnostic laboratory of the Avian Diseases Department, Faculty of Veterinary Medicine, University of Tehran, from different parts of Iran. The PCR test was performed on all samples to identify BFDV positive cases. Specimens were then selected for DNA sequencing from each species. In some cases, while the presence of PBFD had been suspected, samples from clinically healthy birds were also examined for the presence of BFDV. All samples were stored in a freezer set at −20°C to await further molecular assessment.

### DNA extraction and polymerase chain reaction

In this study, DNA was extracted from feathers as described by Taberlet and Bouvet (33) and Morin et al. (34), with a few modifications. To obtain a sample from a feather, a sterilized scalpel blade was used to chop a portion of feather pulp measuring approximately 5 mm in length, followed by washing in 70% ethanol, then in sterile water, before transferring it to a sterile microfuge tube containing 500 ml of lysing buffer [4 mM KCl, 10 mM Tris-HCl pH 8.0, 2.5 mM MgCl_2_, 0.5% Tween 20, 0.5% Nonidet P40 containing proteinase K (250 mg/ml)] and incubating it at 37°C for 1.5 h. The keratinous sheath material became opaque after being digested by proteinase K, and by centrifuging the solution, undigested material was removed, and the solution was then heated for 10 min at 95°C to inactivate proteinase K. All these materials were provided from SinaClon (Tehran, Iran). The DNA was extracted from the supernatant fluid using an animal tissue DNA isolation kit (Denazist, Tehran, Iran) following the manufacturer's recommended procedure. Mock extractions were performed in parallel as a control for cross-contamination of viral DNA. The extracted DNA was stored at −80°C awaiting further use.

For PCR, a 717 bp fragment of the BFDV ORF V1 (*rep* gene) was amplified with primer sense 5′- ACCCTACAGACGGCGAG-3′ and primer antisense 5′-TCACAGTCCTCCTCCTTGTACC-3′ (35) in a 20-μl reaction volume consisting of 10 μl of PCR Master Mix (0.25 U/μl *Taq DNA polymerase*, 2x PCR buffer, 0.4 mM dNTPs, 3.2 mM MgCl_2_, 0.02% bromophenol blue), 1 μl (10 μM) of each of forward and reverse primers, 3 μl of extracted DNA template, and 5 μl of PCR-grade water. The amplification was programmed in a thermocycler (SensoQuest, Germany) as follows: 94°C for 5 min followed by 35 cycles of 55°C for 30 s, 72°C for one min, and a final extension of 72°C for 10 min.

All DNA extracts were also amplified by PCR using primers 5′-CAGACGCCGTTTCACAACCAATAG-3′ and 5′-GGGTCCTCCTTGTAGTGGGATC-3′ as forward and reverse primers, respectively, to amplify a 495-nucleotide fragment of the sequence encompassing the putative capsid gene. While the same amplification reaction volume and content described above were used, cycling conditions differed as follows: 50°C for 2 min, 95°C for 10 min, 40 cycles of 95°C for 15 s, 51°C for 30 s, with 1 min of elongation at 60°C at the end. DNA of confirmed BFDV available in our laboratory and a reaction mixture without a DNA template were used as positive and negative controls, respectively. All samples were run in duplicate and in random order, and amplified products were detected by gel electrophoresis in 1.5% agarose gel in 1x TAE buffer with the addition of DNA Safe Stain^®^ (SinaClon) and visualized under UV illumination. All primers of this study were synthesized by Bioneer (South Korea), and other materials used in PCR reactions were provided from SinaClon.

### Data analysis

Prevalence-quantification analysis was performed in the screening process of all collected samples, infected or uninfected, to assess the prevalence of BFDV in the psittacine population of Iran. The prevalence rate was calculated using the simple equation: (Number of positive BFDV tests/total tests)×100, and the number of positive BFDV test results was counted for each individual species. Additionally, a geographic analysis within the samples' inventories was performed to further understand the geographical distribution of psittacine birds in the country. Based on this analysis, 898 inventories include the living location of the bird and 197 samples were submitted with unknown living location. Therefore, a geographic distribution analysis was performed on the basis of this information.

### Sequence and phylogenetic analysis

To obtain the sequence data for analysis, 17 samples (10 samples for the *cap* gene and seven samples for the *rep* gene) were selected for DNA sequencing. Using PCR primers as sequencing primers, PCR products were purified using the Roche purification kit (Roche Molecular Biochemicals, Mannheim, Germany) and submitted for automated sequencing in both directions at the Genfanavaran Co. (Tehran, Iran). The sequence data were constructed using the Chromas program and aligned using the Muscle function of MEGA version 11 (36). An alignment search tool (BLAST) was used to analyze the sequences for phylogenetic analysis, and for comparison purposes, entries available in the GenBank were used. To construct the phylogenetic tree, a neighbor-joining tree representing 1,000 bootstrap replications was used in both *rep* and *cap* sequences. It should be noted that all phylogenetic and genetic analysis was performed at P-distance mode on both nucleotide and amino acid substitution types, and all sequence data were submitted to the GenBank database using the assigned accession numbers shown in Table 1.

**Table 1:** Detailed information of the 17 beak and feather disease virus (BFDV) field isolates characterized in this study.

**Protein type**	**Sequence ID**	**Isolate**	**Host**	**Accession no**.
Replication-associated protein	seq1	BFDV-1-SD-IR-Lovebird	*Agapornis fischeri*	OP039995
	seq2	BFDV-2-SD-IR-Cockatiel	*Nymphicus hollandicus*	OP039996
	seq3	BFDV-3-SD-IR-Cackatoo	*Cacatua galerita*	OP039997
	seq4	BFDV-4-SD-IR-Ringneck parakeet	*Psittacula krameri*	OP039998
	seq5	BFDV-5-SD-IR-Budgerigar	*Melopsittacus undulatus*	OP039999
	seq6	BFDV-6-SD-IR-Cockatiel	*Nymphicus hollandicus*	OP040000
	seq7	BFDV-7-SD-IR-Lovebird	*Agapornis fischeri*	OP040001
Putative *cap*sid protein	seq8	BFDV-8-SD-IR-Lovebird	*Agapornis fischeri*	OP039985
	seq9	BFDV-9-SD-IR-Cockatiel	*Nymphicus hollandicus*	OP039986
	seq10	BFDV-10-SD-IR-Cackatoo	*Cacatua galerita*	OP039987
	seq11	BFDV-11-SD-IR-Ringneck parakeet	*Psittacula krameri*	OP039988
	seq12	BFDV-12-SD-IR-African gray parrot	*Psittacus erithacus*	OP039989
	seq13	BFDV-13-SD-IR-Monk parakeet	*Myiopsitta monachus*	OP039990
	seq14	BFDV-14-SD-IR-Conure	*Pyrrhura molinae*	OP039991
	seq15	BFDV-15-SD-IR-Budgerigar	*Melopsittacus undulatus*	OP039992
	seq16	BFDV-16-SD-IR-Cockatiel	*Nymphicus hollandicus*	OP039993
	seq17	BFDV-17-SD-IR-Alexandrine parakeet	*Psittacula eupatria*	OP039994

### Genomic analysis

To investigate the genomic aspects of the BFDV, the rep and cap sequences were used to characterize the gene evolutionary probability, amino acid substitution rate during cell replication process, and frequent codon usage analysis, and lastly the new variant analysis. To this end, all sequences that previously used for phylogenetic analysis, were then genetically aligned using the “*muscle*” alignment mode. In follows, the method of each gene evaluation is described in detail.

### Evolutionary probability

Based on the long-term substitution patterns captured in multiple sequence alignment, EP represents the independent evolutionary expectation of observing a variant in a host population, with an evolutionary probability value higher than 0.05 representing the evolutionary permissible (ePerm; EP ≥ 0.05) and forbidden (eForb; EP < 0.05) variants (37). To this end, sequence evolution was examined by analyzing the *rep* and *cap* sequences of BFDVs to determine their evolutionary likelihood using MEGA version 11 (36). To this respect, the Jones-Taylor-Thorntone (JTT) method was used to determine the EP value for each possible variants within both rep and cap nucleotides and amino acids genetic structure.

### Substitution rate

Generally, nucleotide substitutions occur in DNA sequence over evolutionary time in gene replication process (38). As a consequence, the number of nucleotides' changes, also known as substitution rate, alters the codon positions of DNA sequence that lead to different amino acids being produced. Note that the EP represents the independent evolutionary expectation of observing a variant in a host population, based on the long-term substitution patterns captured in multiple sequence alignment. To this end, the amino acid substitution rate was determined by using the Jones Taylor Thornton (JTT) model within MEGA software using the phylogeny tree and maximum likelihood statistical method.

### Codon analysis

According to general knowledge, codons are composed of three adjacent nucleotides that are involved in the coding of amino acids (39). The arrangement of the three nucleotides changes during evolutionary time as a result of nucleotide substitutions (39). The codon analysis is therefore necessary in order to determine the basis for the mutation in a gene. To this end, relative synonymous codon usage (RSCU) is calculated to determine the characteristics of synonymous codon usage from a sequence without being influenced by the amino acid composition and coding sequence size of sequence. By using MEGA software (36), the RSCU value of the rep and cap sequences was determined based on Equation 1:


(1) $$\begin{array}{l} \text{RSCU}=\text{S}\times \frac{{\text{N}}_{\text{c}}}{{\text{N}}_{\text{a}}} \end{array}$$

Where,

S = the number of synonymous codons encoding the same amino acid,

N_c_ = the frequency of the codon in the genome, and

N_a_ = the relative frequency of the codon for the that amino acid.

### Variant analysis

As part of the genomic analysis, a variant analysis was conducted to identify new variants of BFDV within endemic populations of psittacine birds. To accomplish this, two groups of sequences were identified as suspected and reference sequences. Sequences in the suspected group have a greater nucleotide distance from the closest public sequence in GenBank. A phylogenetic tree analysis was conducted first, followed by a nucleotide distance analysis to identify the rep and cap suspect groups. Conversely, the reference group is comprised of the sequences that are included in the phylogenetic tree, excluding the suspected sequences. Two of these groups were defined using DNA alignment using MEGA software. As a result, the mean distance between groups was calculated using 1,000 bootstrap replications considering all three codon positions.

## Results and discussion

### Prevalence data analysis

The BFDV distribution within Iran's endemic population of psittacine birds shown in Table 2, summarizes the results of the observations and BFDV testing over 1,095 samples, including positive and negative results. Table 2 indicates that 58 percent of all studied species were infected with the BFDV virus. It appears that this disease is endemic in Iran among psittacine birds based on such an outbreak within a population of 1,095 individual birds. In addition, it should be noted that the infection rates presented are for all birds, regardless of the severity of the disease. It can be seen in the table that lovebirds and cockatiels comprised the majority of the BFDV positive cases (64%) followed by budgerigars (63%). It can be also noted that, based on the number of observations for these three species, their populations are high in Iran, so the probability of new BFDV variants emerging within these species would be higher than for other species. A possible explanation for this could be the high rate of BFDV migration among high-population species within a clone, resulting in less local adaptation of the virus. This could cause an increase in the variation of the virus gene, resulting in a potential virus mutation (40). Based on further analysis of the observations, there were species for which the BFDV had not yet been observed within the collected samples, including Amazon parrots, galahs, toucans, and rosellas. Despite the lack of evidence of BFDV within these species, due to their small population, it is not possible to conclude that they are not at risk from BFDV, and further sample collection and testing would be necessary to determine the level of threat posed by BFDV in such species. A second point of observation was related to species with small observations and high BFDV infection rates. For example, cockatoos and Rainbow lorikeets exhibited BFDV positive rates of 42%, while the number of samples of these species was small in comparison with other species. It should be noted that Razmyar et al. (41) documented the first observation of BFDV in Iran while examining a cockatoo showing some clinical symptoms. In their opinion, the bird had been infected with a horizontal mode of the virus prior to being imported into Iran. In the same page, Ghorani et al. (42) reported that BFDV existed in more than 35% of cockatiel populations of Iran. All the studied samples were provided by a bird market in Isfahan province, located in center of Iran. They believed that the exchange and trade of the infected host is the main reason for spreading the BFDV virus among Iranian cockatiel populations.

**Table 2:** Results for PCR screening of different species for the beak and feather disease virus (BFDV).

**Bird scientific name**	**Common name**	**Test results**		**BFDV rate, %**
		**Negative**	**Positive**	
*Agapornis fischeri*	Lovebird	53	93	64
*Amazona farinosa*	Amazon parrot	2	0	0
*Ara ararauna*	Macaw	6	3	33
*Aratinga solstitialis*	Sun parakeet	7	2	22
*Cacatua galerita*	Cockatoo	7	5	42
*Eolophus roseicapilla*	Galah	2	0	0
*Melopsittacus undulatus*	budgerigar	15	26	63
*Myiopsitta monachus*	Monk parakeet	18	29	62
*Nymphicus hollandicus*	Cockatiel	178	320	64
*Adelaide Rosella*	Rosella	5	0	0
*Poicephalus senegalus*	Senegal parrot	5	1	17
*Psittacula eupatria*	Alexandrine parakeet	37	19	34
*Psittacus erithacus*	African gray parrot	45	54	55
*Psittacus timneh*	Timneh parrot	3	0	0
*Pyrrhura molinae*	Green-cheeked parakeet	87	76	47
*Ramphastos toco*	Taco toucan	2	0	0
*Trichoglossus haematodus*	Rainbow lorikeet	7	5	42
*Total*	All studied species	462	633	58%

Further analysis of Table 2 data indicates that since Senegal parrots, macaws, and Sun parakeets, all with lower sample availability, exhibit a low rate of BFDV ranging from 17 to 33%, it can conceivably be concluded that more than twelve species of psittacine birds in Iran have been found to be susceptible to BFDV infection, and in seven of these species BFDV infected more than 40% of cases. It was also understood that, although the size of bird populations of these species was low, the treat of BFDV evolution still exists, especially over long periods of time. From the epidemiological point of view, species like lovebirds, budgerigars and cockatoos with larger population size are more susceptible to BFDV due to possibility of vertical and horizontal infection modes. This view was previously taken by Ritchie et al. (18) in which the authors expressed the belief that despite genetic variants remaining neutral, population-size variation would accentuate divergence among isolated populations. From another standpoint, Woods and Latimer (43) believed that the likelihood of vertical transition is particularly high during the breeding season. In the same vein, there is a possibility that BFDV sequence evolution may occur during the breeding season (44, 45); this cannot be ruled out for lovebird, budgerigar and cockatiel since their population size is large. It was also indicated that, despite the small population of Rainbow lorikeet, there was a high BFDV rate among these species, perhaps due to conditions under which the Rainbow lorikeet can live with large groups of other birds in the wild (46). This tendency may suggest the opportunity to keep them in the same bird nursery with other types of psittacine birds such as lovebirds, but in such a case, coevolution of BFDV may be of concern. Since there are many wholesale markets where different species are kept and sold together, the possibility of coevolution in the population of Psittaciformes in Iran cannot be denied. In such markets, even if no infected birds are present, since a variety of birds may have previously inhabited the area, birds kept in this way are at high risk of BFDV infection and coevolution. In a study conducted by Amery-Gale et al. (10), it was proven that when a new host occupies a nest of a previously-infected bird, BFDV can be transmitted to the new host.

**Figure 1:**
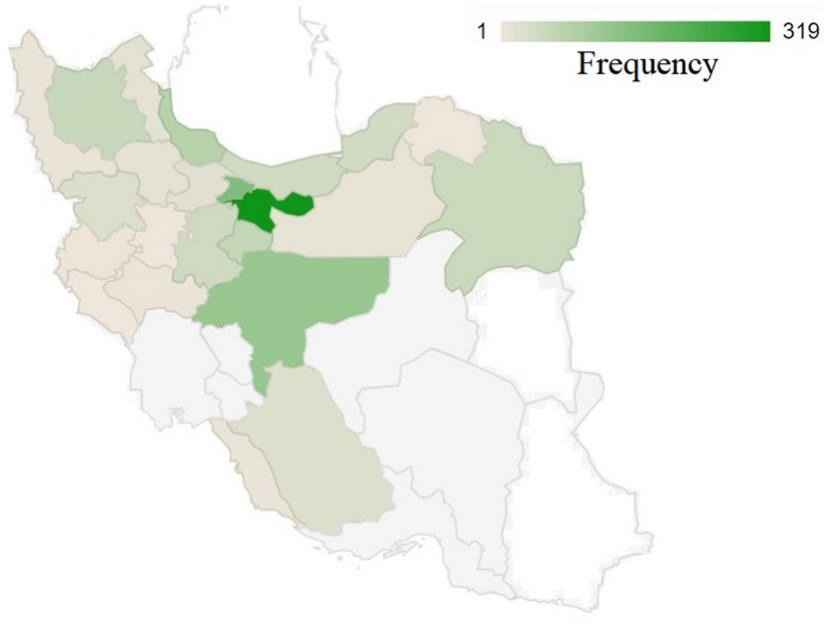
The geographical distribution of collected samples across 31 provinces of Iran. A color intensity scale indicates the geographical density; darker colors indicate a higher density.

Figure 1 illustrates the distribution of studied birds throughout the country. The greatest number of samples were collected from Tehran Province followed by adjacent provinces, which are located in the center to northern parts of the country. There might be a number of factors contributing to the high psittacine bird population in these areas, including population density and the presence of breeding centers and bird national parks. Additionally, this figure shows that the lowest number of specimens were collected from the country's eastern and southeast regions. This may be due to the geographical characteristics of these regions, which are primarily composed of desert with a very low population density. And also, the presence of other bird hospitals in those areas where may receive the samples from adjacent provinces. Conversely, from west to center and north, there is a relatively higher density of received samples. The reasons for this may be related to several factors: 1) the density of the population, 2) the presence of national parks, and 3) the opportunity for wild birds to migrate to this area as a result of the presence of the national forest and rich habitat.

In a study conducted by Fogell et al. (5), a considerable BFDV infection rate was observed from wild to captive birds in Australia, southern part of Asia, and South Africa. Moreover, epidemiological linkages and viral movement patterns shows that the European countries are like a hub for circulating the BFDV originated from Australia, east Asia, south Africa, and American continent (47). It appears that the role of countries that import psittacine bird is significant in the global dissemination of BFDV and accelerating genetic diversification (47). Since many psittacines have a foreign origin, the role of importation of those is stressed in Iran. Additionally, as many wild parrots inhabit the northern and northwestern forests of Iran, the incidence and transmission of the BFDV by wild toxic birds are likely to pose a threat to domestic psittacine bird populations owing to the presence of green land in the surrounding countries such as Türkiye, Georgia, Turkmenistan, and Armenia which are close to the European countries.

### Phylogenetic analysis

#### Phylogenetic tree

**Figure 2:**
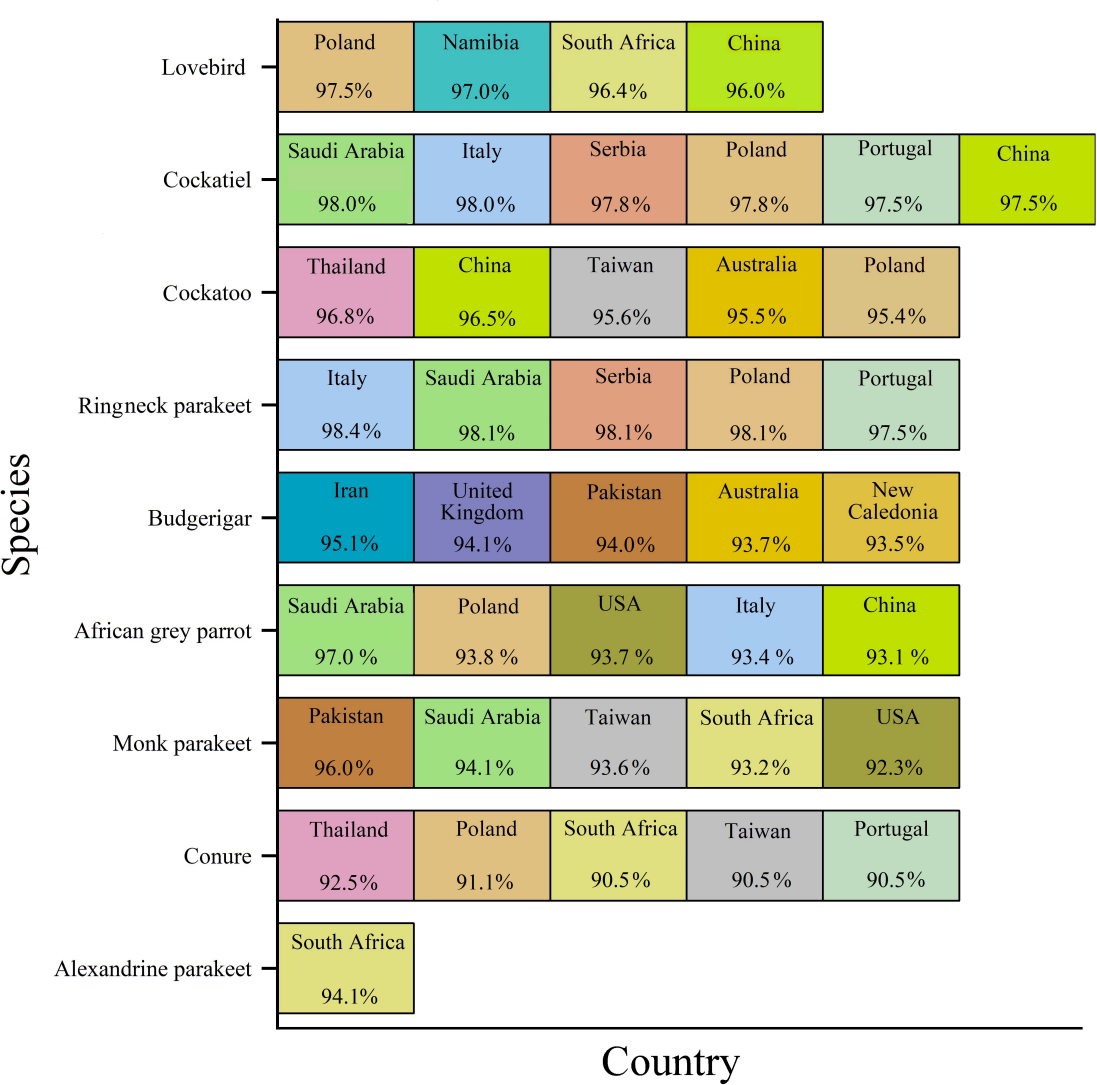
GenBank-published BFDV sequences from different countries and species. Countries were arranged from left to right based on percentage of similarity, with the leftmost showing the greatest similarity.

The geographic roots of the sequences available in GenBank used for analysis will be discussed before describing the phylogenetic analysis. As shown in Figure 2, each species was associated with a couple of countries. Only sequences with similarities to the BFDV-infected hosts >90% were compared with our data. The countries were arranged from left to right based on percentage of similarity, with the left one corresponding to the greatest similarity. Cockatiels, ranked as a psittacine bird with the greatest diversity of origin, exhibited high similarity to GenBank-published sequences from six different countries; it shared the greatest similarity with sequences from Saudi Arabia, followed by sequences from four European countries, and finally with sequences from China (Figure 2). Similarity of lovebird sequences to the Polish sequences was also noteworthy. The African gray parrot sequences shared the highest similarity with those of Saudi Arabia, with a slight difference in the order in which the similarity with other countries follows. There was a high degree of similarity between the cockatoo and conure sequences with those from Thailand. An interesting aspect of this chart pertained to the Alexandrine parakeet that exhibited the greatest similarity to sequences from South Africa. According to this chart, sequences from budgerigar populations showed the highest similarity with sequences reported from Iran, followed by sequences reported from the United Kingdom, Pakistan, Australia, and New Caledonia. In Iran, there was a relatively large population of budgerigars. Finally, sequences from the Monk parakeet had the highest similarity with sequences reported from Pakistan, followed by sequences reported from Saudi Arabia, Taiwan, South Africa, and the United States. In addition, sequences from Saudi Arabia, South Africa, Poland, and China were similar to sequences from all species except for budgerigars. The BFDV, therefore, appears to be more widespread globally among most studied species except for the lovebird and the Alexandrine parakeet, and the virus within the endemic population of species originated from a variety of countries, mainly from Europe and South Africa and some East Asian countries. In comparison to all others, Alexandrine parakeets shared one origin similarity, suggesting that it could have been imported directly from South Africa. Upon further investigation in literature, it was revealed that the Alexandrine parakeet was once among the world's rarest parrots (12). Therefore, another explanation for the single origin similarity of this bird lies in its low population size. Overall results suggest a couple of infection waves resulting from international trading of exotic birds. To support this idea, Franzo et al. (48) claim that BFDV was predominantly transferred from wild to domestic populations, highlighting the role of a bird-trading market. A study conducted in Iranian cockatiel populations, showed that the Iranian isolates have the most similarity to Saudi Arabian isolates (42).

**Figure 3:**
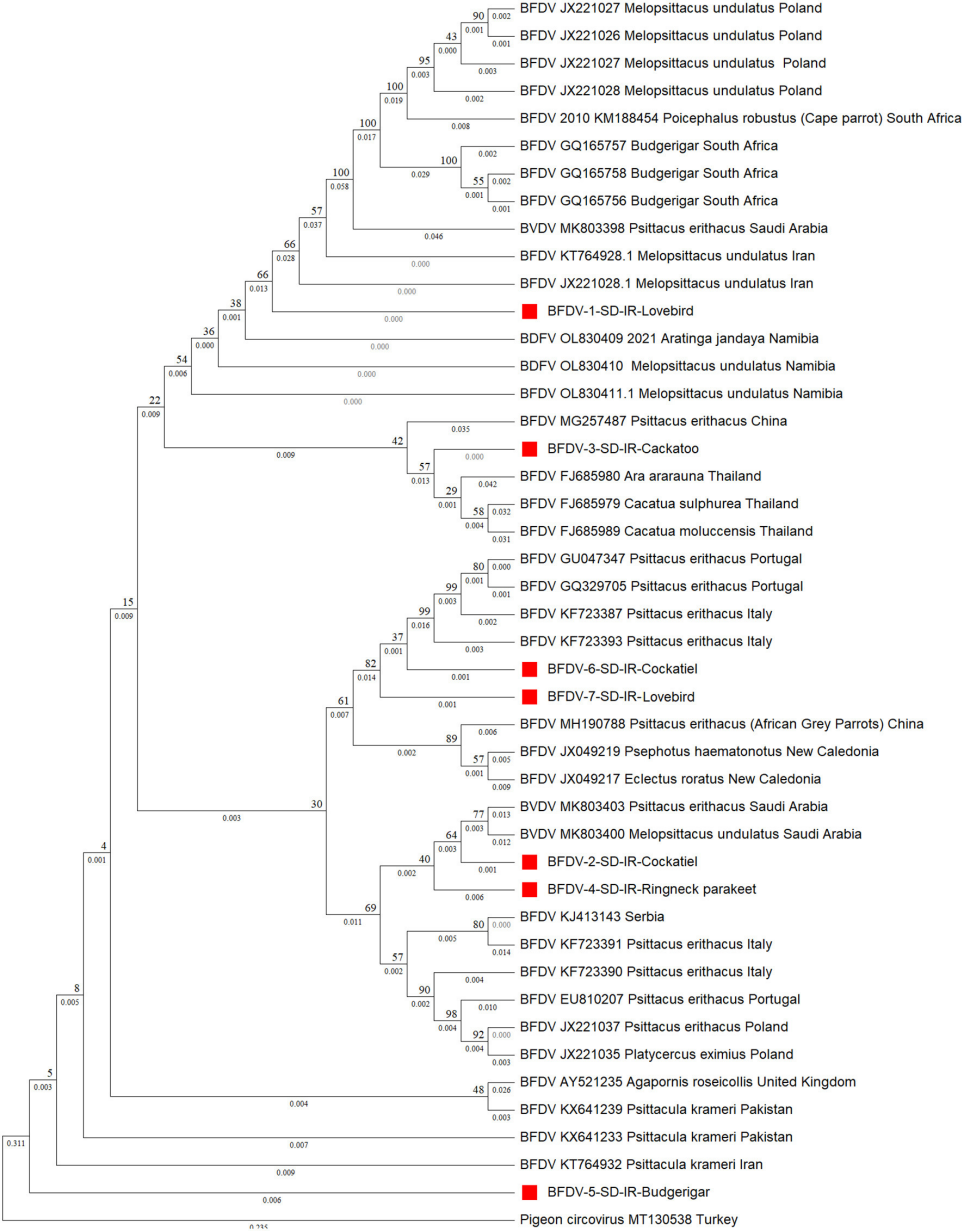
Neighbor-joining phylogenetic tree constructed using Mega ver. 11 at 1,000 number of bootstrap replications on *rep* sequences, demonstrating distance of the infected hosts (OP039995-OP040000) in relationship to public BFDV *rep* sequences from other Psittaciformes available in GenBank. Numbers at nodes *rep* represent the frequency of Bayesian posterior probability between two subsequent clusters or strains. Each sequence labeled with BFDV year of specimen collection, GenBank accession numbers, host species, and country. The samples used in this study are marked with a red color cubic (■) symbol.

Turning to phylogenetic analysis, *rep* and *cap* neighbor-joining phylogenetic trees were constructed using seven and 10 BFDV obtained sequences, respectively. In total, 36 *rep* and 26 *cap* GenBank-published BFDV sequences were used along with this study's sequences for phylogenetic analysis. GenBank sequence selection was based on a minimum pairwise-similarity threshold of 90%. Figure 3 is a *rep* phylogenetic tree containing information on cluster and distances. To validate the analysis, an outgroup sequence related to a pigeon (MT130538) was added to the tree and, as shown, it has the highest distance from all other species (0.546). It can also be seen that a lovebird sequence was placed within a cluster of subfamilies from South Africa and Poland. One exception is another lovebird *rep* sequence that was placed as an out branch of the cluster containing subfamily species from Italy (KF723387) and Portugal (GU046347), conceivably attributable to endemic BFDV evolution within the lovebird population since it has a higher branch distance compared to its counterparts. Such an explanation previously introduced by Massaro et al. (49), expressed the belief that higher genetic potential for evolution exists within captive-breeding facilities that may provide opportunities for horizontal transmission of diseases; as indicated, a cockatoo placed within a cluster with a common ancestor with Thailand counterparts (FJ685979-80) was observed as a sister cluster to one containing a lovebird. It also exhibits lineage with a large cluster that included the other species except for budgerigar. Figure 3 clearly also shows that the budgerigar was an outbranch from all other ancestor BFDV, with a distance branch length of 0.09 from the other common BFDV ancestors and species. Budgerigar can be categorized as a host with high potentiality for virus mutation in which three new variants within population of this bird have been reported in China and Japan (30).

**Figure 4:**
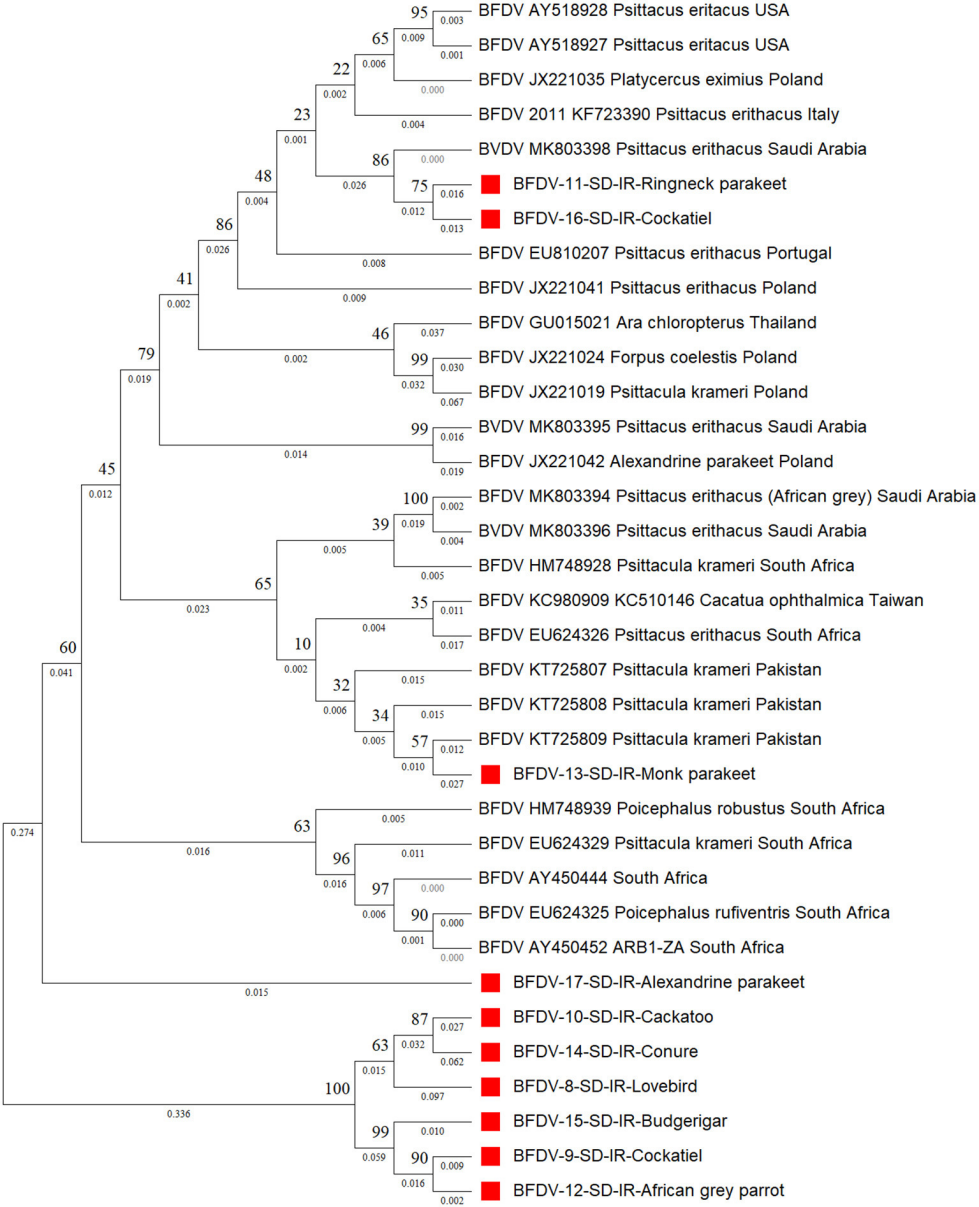
The neighbor-joining phylogenetic tree constructed on *cap* sequences using Mega ver. 11 and 1,000 bootstrap replications. This tree illustrates the distance between the infected hosts (OP039985- OP039994) and public BFDV *cap* sequences from other Psittaciformes available in GenBank. Numbers at nodes represent the frequency of Bayesian posterior probability between two subsequent clusters or strains. Each sequence labeled with BFDV year of specimen collection, GenBank accession numbers, host species, and country. The samples used in this study are marked with a red color cubic (■) symbol.

Figure 4 shows the *cap* phylogenetic tree related to the 10 *cap* DNA sequence of infected birds. As this figure shows, except for the Monk parakeet, the cockatiel and the Red-neck parakeet, other *cap* sequences had a weaker support from their peers, suggesting that the mutation rate of the *cap* sequence is greater than that of *rep*. This analogy is also supported by a study conducted by Sarker et al. (50), in which a similar finding was supported by comparing the mutation rate of *rep* and *cap* sequences of same parrot species. However, there is no solid evidence to explain the reason for high divergence within the *cap* protein responsible for weaker conservation through BFDV transmission within host species. In detail, six out-branches were found within the cloned *cap* sequences in which two of those were previously noticed by *rep* phylogenetic tree analysis that included budgerigars and lovebirds. Cockatiel was also a sister to the Ringneck parakeet, and both were in a cluster sister with an isolate from Saudi Arabia (MK803398). Furthermore, although the Alexandrine parakeet was located close to the six out-branch sequences (Sequence ID: 8-10, 12, 14, 15, and 17), it was supported by a strongly shared ancestor from South Africa (HM748939). Such a high similarity of Alexandrine parakeet could be related to the conservation management that countries applied to this certain type of parrot for recovery (12). Therefore, the coevolution threat for this bird is low compared to others.

There are, however, other factors that can affect BFDV infection and mutation, including population size, conditions of birds both in captivity or in the wild, and the international trade in exotic birds. Comparison between the *rep* and *cap* pairwise distances supported the low divergence of the *rep* sequence, as previously asserted by Sarker et al. (50). This might be related to the ultra-conserving ability of the *rep* sequence compared to the *cap* sequence; as described below, analysis of nucleotide pairwise distance supported this hypothesis.

### Pairwise distance analysis

**Figure 5:**
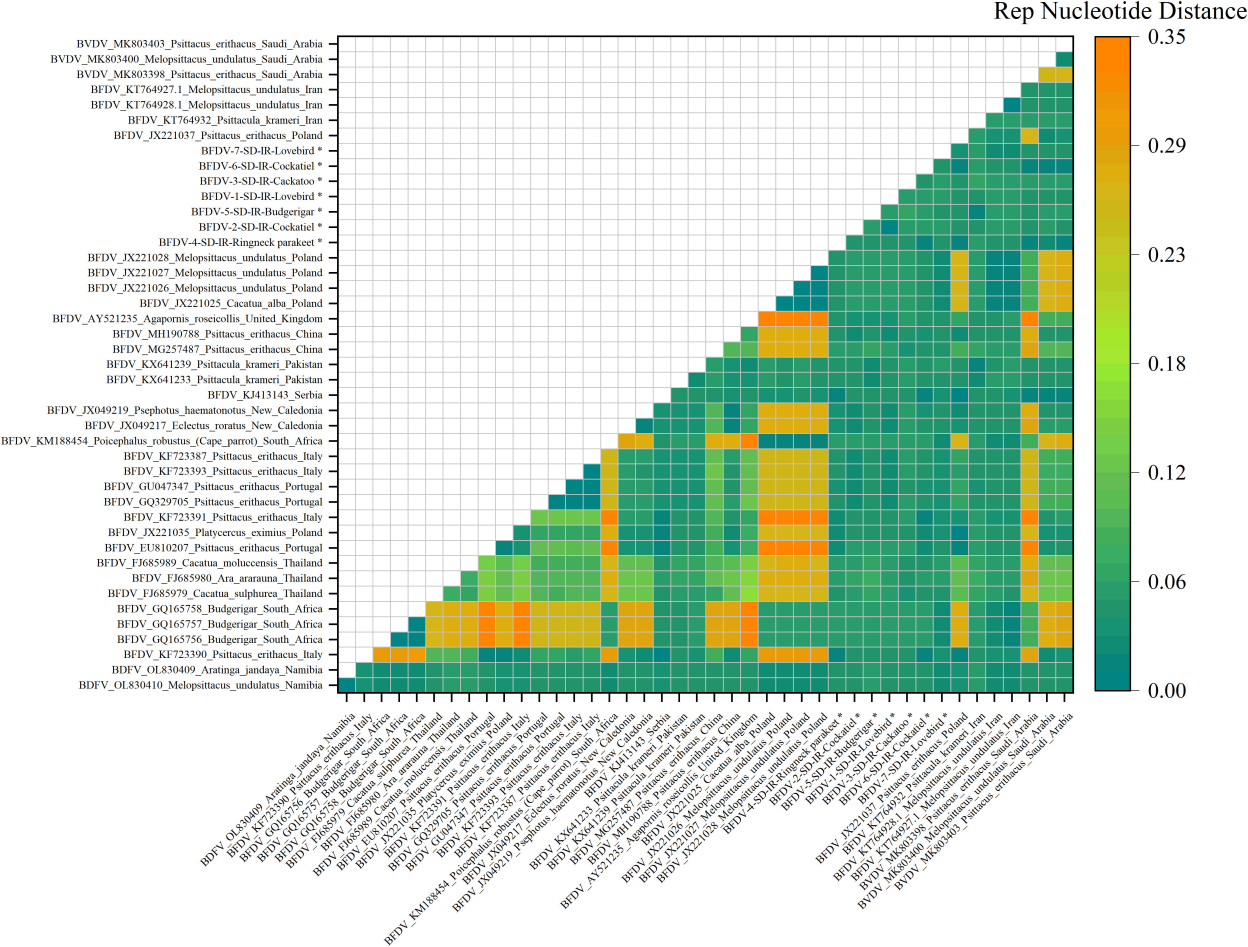
An illustration of the pairwise distance between *rep* nucleotide sequences based on the pairwise deletion of gaps in all seven *rep* and 36 GenBank sequences. The labels represent the sequence information within GenBank with year of sample collections, GenBank accession number, the name of bird, and the country of host. All *rep* sequences of this study are marked with a black star (*).

**Figure 6:**
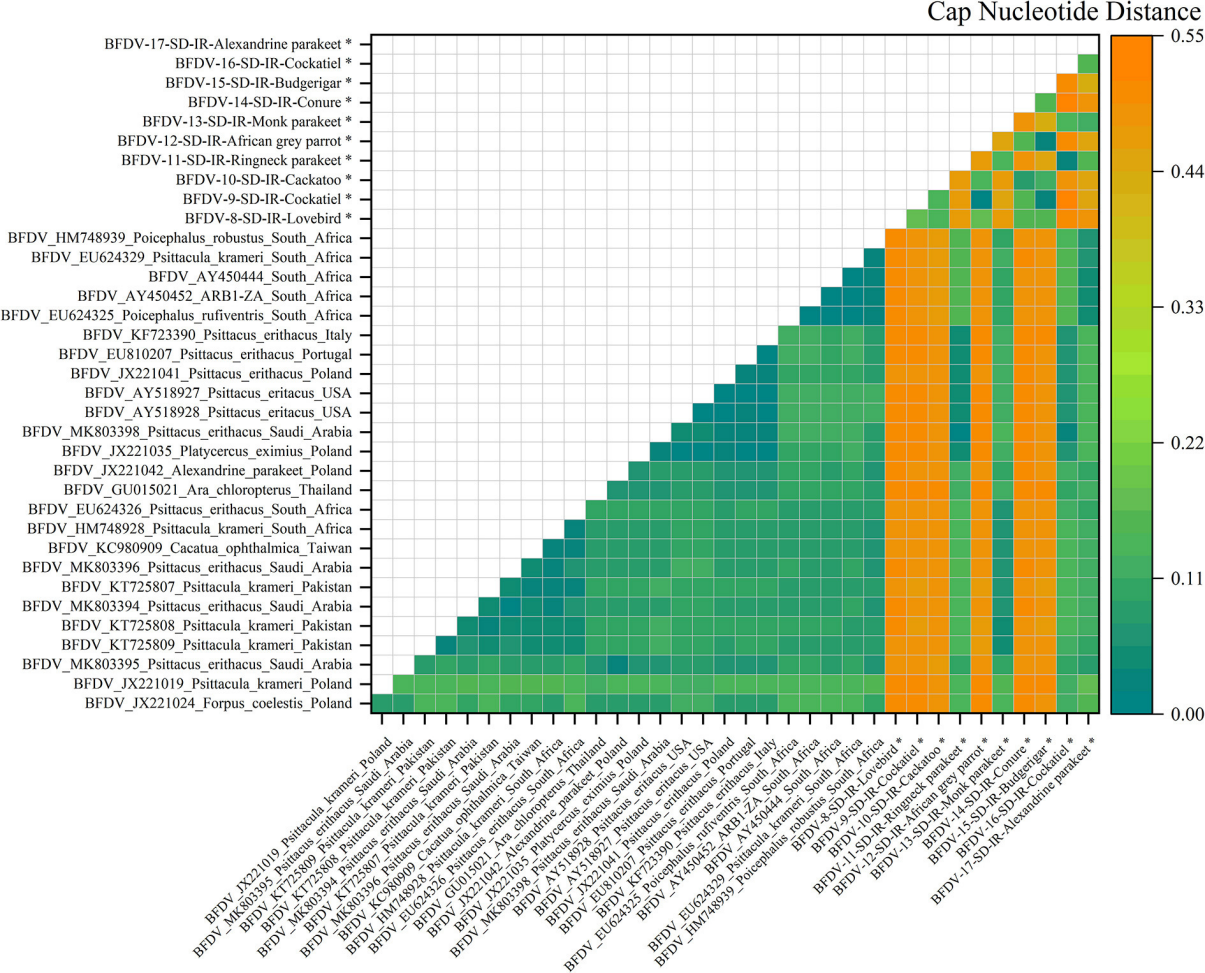
An illustration of the pairwise distance between *cap* nucleotide sequences based on the pairwise deletion of gaps in all ten *cap* sequenced sequences and 26 PubMed sequences. The labels represent the sequence information within GenBank with year of sample collections, GenBank accession number, the name of bird, and the country of host. All *cap* sequences of this study are marked with a black star (*).

Pairwise distances shown in in Figures 5, 6 represent further analysis of phylogenetic trees. As shown in Figure 5, all *rep* sequence nucleotides of seven BFDV infected hosts of this study reflected a distance <0.15, with budgerigar having at least 85% identity (or a distance of 0.15) with most of the GenBank-published sequences. Among all the seven hosts, the lovebird sequences exhibited no pairwise distance smaller than 0.06, suggesting that lovebirds may not be affected by infection waves of BFDV due to international trading with other countries. Figure 5 also shows some large nucleotides distances (> 0.2) that in GenBank were all attributed to pairwise distances of relevant sequences. Further analysis of Figure 5 revealed that the closest subfamily of the Ringneck parakeet was *Psittacus erithacus* located in Italy. Since it had a distance <0.03, BFDV transmission seems the most plausible source of infection in the Ringneck parakeet. The infected cockatoo host also exhibits a small pairwise distance with a subfamily from Thailand (FJ685979-80-89), and since Thai variants exhibited small distances from most of the infected studied hosts, it can be stated that, except for the lovebird, the prevalence of the Thai species on the endemic hosts is predominant.

Figure 6 illustrates the nucleotide pairwise distance for all *cap* sequences of 10 infected hosts, and compared to the *rep* pairwise distance, *cap* showed a broader range of distance between the two peer sequences. It can be conclusively stated that the *cap* sequence exhibits more divergence than *rep*, possibly related to the high conserving ability of the *rep* sequence compared to that of the *cap* sequence; the analysis of nucleotide pairwise distance is supported by the hypothesis previously stated by Sarker et al. (50). As can be seen, six infected sequences exhibited the highest distance from its peer hosts, including cockatoo, African gray parrot, cockatiel, lovebirds, conure, and budgerigar. This was also visible in the *cap* phylogenetic tree that were all in out-branch locations of other hosts. Further exploration demonstrates that Ringneck parakeet has almost less distance with most of the relevant sequences in GenBank, and the same observation could be seen for cockatiel. Both examples reflect very small distances with hosts from Saudi Arabia (MK803398). The Alexandrine parakeet also exhibited the highest similarity with South African hosts (HM788939, AY450444-52, and EU624325-29), while the Monk parakeet shared some identity with hosts from different countries, including Pakistan, Saudi Arabia, South Africa, and Taiwan. According to a study conducted by Varsani et al. (30), a 0.06 distance (94% pairwise identity) has been introduced for strain demarcation of BFDV. Based on this threshold, combined with the analysis for the *rep* and *cap* pairwise distance, there appears to be a possibility of BFDV mutation within endemic hosts.

### Genomic analysis

#### Evolutionary probability

**Figure 7:**
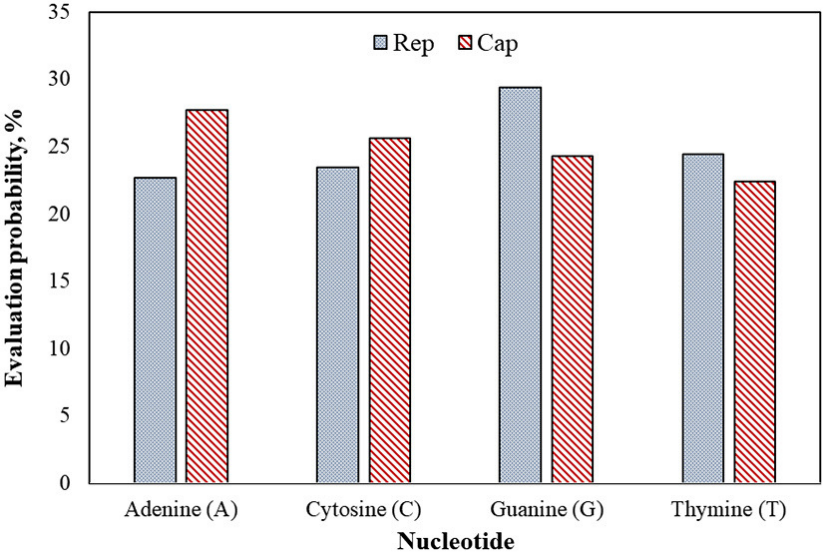
Evolutionary probability of *rep* and *cap* nucleotides from the database used in phylogenetic analysis, including 43 *rep* and 36 *cap* sequences. Mega ver. 11 was used for this analysis based on Tamura-Nei model and Bayesian/Realtime statistical method. All codon positions were considered in this analysis.

**Figure 8:**
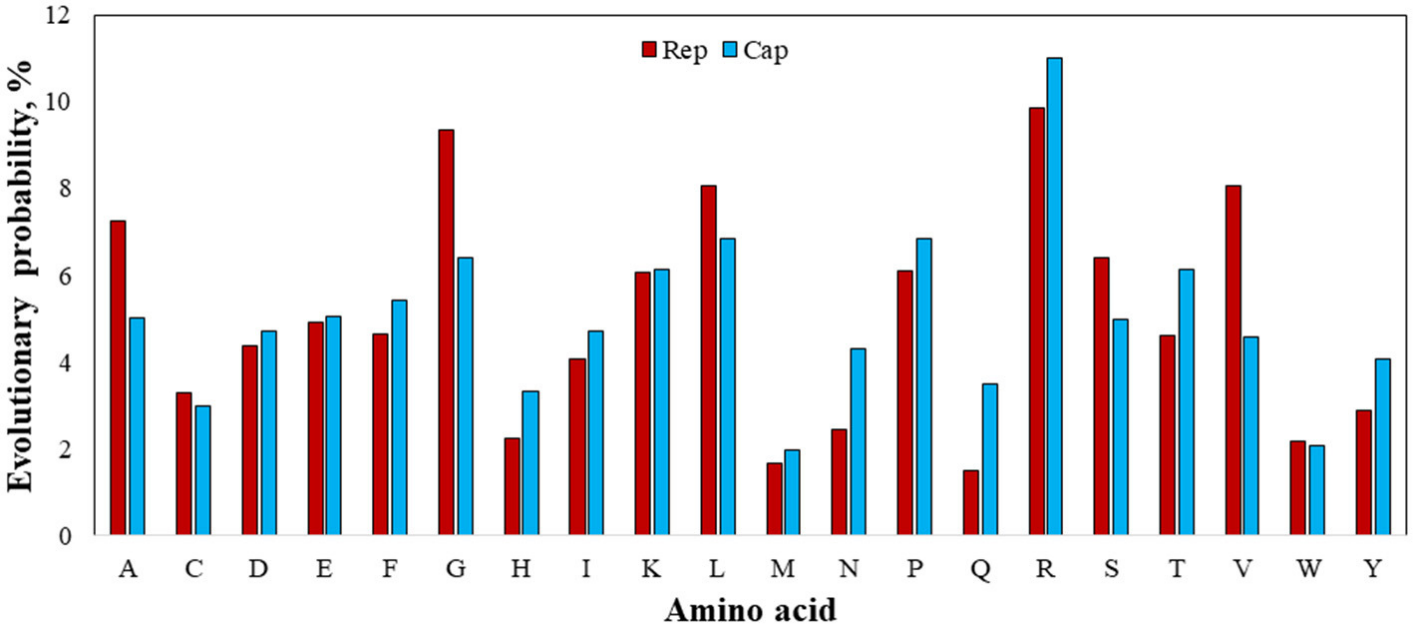
Evolutionary probability of involved amino acids within *rep* and *cap* sequences, including GenBank sequences and *rep* and *cap* sequences. Mega ver. 11 was used for this analysis based on Jones-Taylor-Thornton (JTT) model and Bayesian/Realtime statistical method.

Figure 7 shows that guanine (G) nucleotide had the highest evolutionary probability in *rep* groups, following thymine (T), cytosine (C), and adenine (A), so the G nucleotide had the highest number of transitions and transversion substitutions in the *rep* sequence for the positive BFDV hosts. Since analysis of the *cap* sequences similarly indicated more A nucleotides to be present within the nucleotide sequence, *cap* sequences contain a greater number of substitutions related to the A nucleotide, while *rep* sequences have a greater proportion of substitutions related to the G nucleotide. Since all EP values were higher than 5%, either of the EP values shown in the *rep* and *cap* sequences suggest that all nucleotides involved in all long-term substitutions have potential for creating new variant. In other words, the high evolution probability of all four engaged nucleotides within either *rep* or *cap* sequences were >5%, suggesting that a probability of substitution change exists through the *replication* process of the genome. For better interpretation of BFDV evolution, EP analysis on *rep* and *cap* amino acid was performed, with the result shown in Figure 8.

Figure 8 shows the EP values for all involved 20 amino acids. Since an amino acid with an EP <0.05 (5%) represents an amino acid positioned within an ultra-conserved substitution pattern (eForb), tryptophan (W), methionine (M), tyrosine (Y), histidine (H), asparagine (N), and cysteine (C) were involved in the eForb group during phylogenetic mutations. Conversely, glycine (G), arginine (R), leucine (L), valine (V), alanine (A), lysine (K), proline (P), and serine (S) were least-conserved amino acids involved in the ePerm group upon their greater EP value shown in Figure 7. Since the *rep* and *cap* sequences were clearly observed to have the same amino acids with slight variations in ePerm, potential amino acids involved in permissible substitution patterns can be detected across the sequence on either Rep or Cap proteins. Careful examination of Figure 8 reveals that it is obvious that arginine (R) was most involved in ePerm, more likely to be involved in BFDV mutations. From another point of view, since the concentration of R in the N-terminal is high (51), the role of arginine within the *cap* sequence in cell nuclear localization would be higher than for *rep*. This finding supports the idea that *cap* proteins, in addition to their role in encapsidation, are increasingly recognized as being important components of the life cycles of circoviruses and other ssDNA viruses.

Taking into account the EP analysis and discussion related to the presence of high divergency of *cap*, the high EP value of adenine (A) rather than thymine (T) replaced by uracil (U) as the complementary nucleotide, might help explain for the high divergency of produced Cap encoded protein. In terms of mutation, arginine (R) is the amino acid most prone to change or substitution for other amino acids, followed by G and L amino acids. Tryptophan (W) and methionine (M), on the other hand, during phylogenetic mutations are largely involved in conserved substitution patterns. In the case of an amino acid affecting viral virulence or pathogenicity of BFDV circovirus, since a higher rate of evolution probability reflects a higher likelihood of mutation (52), a more detailed analysis of the *cap* sequence would be able to explain the higher divergence and distance seen in the phylogenetic analysis.

### Substitution rate

**Figure 9:**
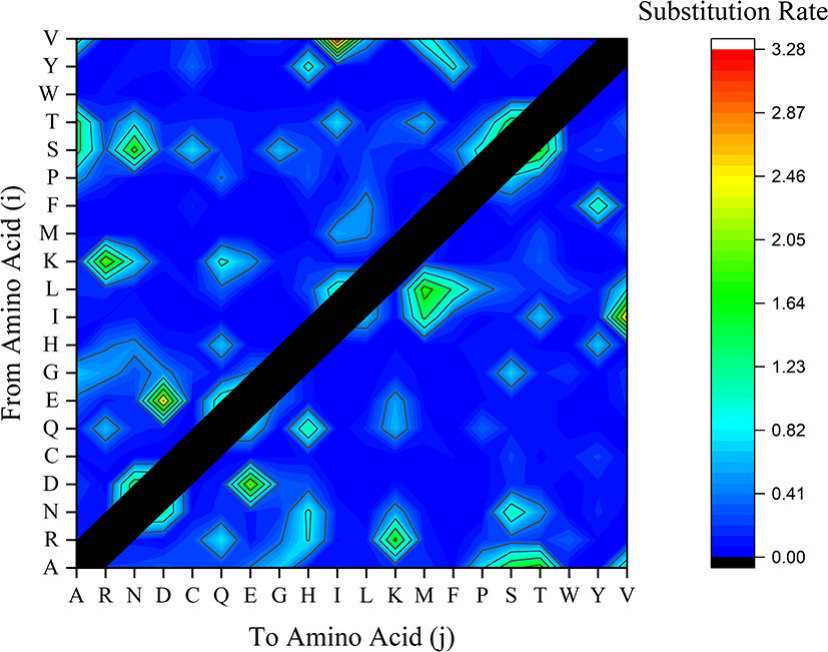
Substituted amino acids rate within BFDV sequences of all *rep* or *cap* sequences including sequences from GenBank and this study. The vertical axle represents the amino acid (i) to be substituted by amino acid (j) as labeled on the horizontal axle.

Another factor contributing to mutation is the substitution rate of potential amino acids. While amino acids R, G, and L have been referred to as the most BFDV amino acids involved in ePerm substitution pattern, the rate of amino acid substitution is also an important factor contributing to gene mutations (53). To investigate this, amino acid substitution rate analysis was performed and the frequency of amino acid conversion from amino acids i to j was represented in terms of substitution rate in Figure 8. This figure shows that the substitution rate of V→ I, E→ D, R→ K, G→ S is high compared to other amino acid substitution. Taking the EP analysis into account, it was found that first three essential amino acid in the BFDV sequence were R, G, and L, and by synchronizing them to amino acid substitution rate, as shown in Figure 9, it was found that R→ K likely makes a greater contribution to virus variation than G→ S. Because both arginine (R) and lysine (K) are ePerm amino acids, the high rate of arginine to lysine amino acid substitution would also highly contribute to BFDV mutation. This could be more predominant in capsid protomers since arginine amino acid was more involved than *rep* in the *cap* transduction process. By analyzing the EP values of the R in the *cap* sequence, it was determined that it had a greater EP value than its counterpart in the *rep* sequence, and further analysis revealed that, despite the high substitution rate of E→ D present in the BFDV circovirus, neither E nor D amino acids were essential in virus evolution, as shown in Figure 8, where both amino acids fell within eForb.

### Codon analysis

The analysis and discussion so far have indicated which amino acids and conversions are most prevalent in BFDV substitution and probably in gene mutation, so the idea of codon position and its significance as a means for linking them to nucleotide positions will next be being discussed. For the purpose of determining which codons were essential to producing R amino acids with the greatest evolutionary potential, all synonymous codons in both *rep* and *cap* sequences were counted and a relative synonymous codon usage (RSCU) was then calculated. When all codons for a particular amino acid are equally used, the RSCU value represents the ratio between the observed usage frequency of one codon in a sequence and the expected usage frequency in the synonymous codon family. It should be noted that codons with RSCU value >1 have positive codon usage bias and were defined as abundant codons, while a RSCU value <1 categorizes a codon as less abundant.

**Figure 10:**
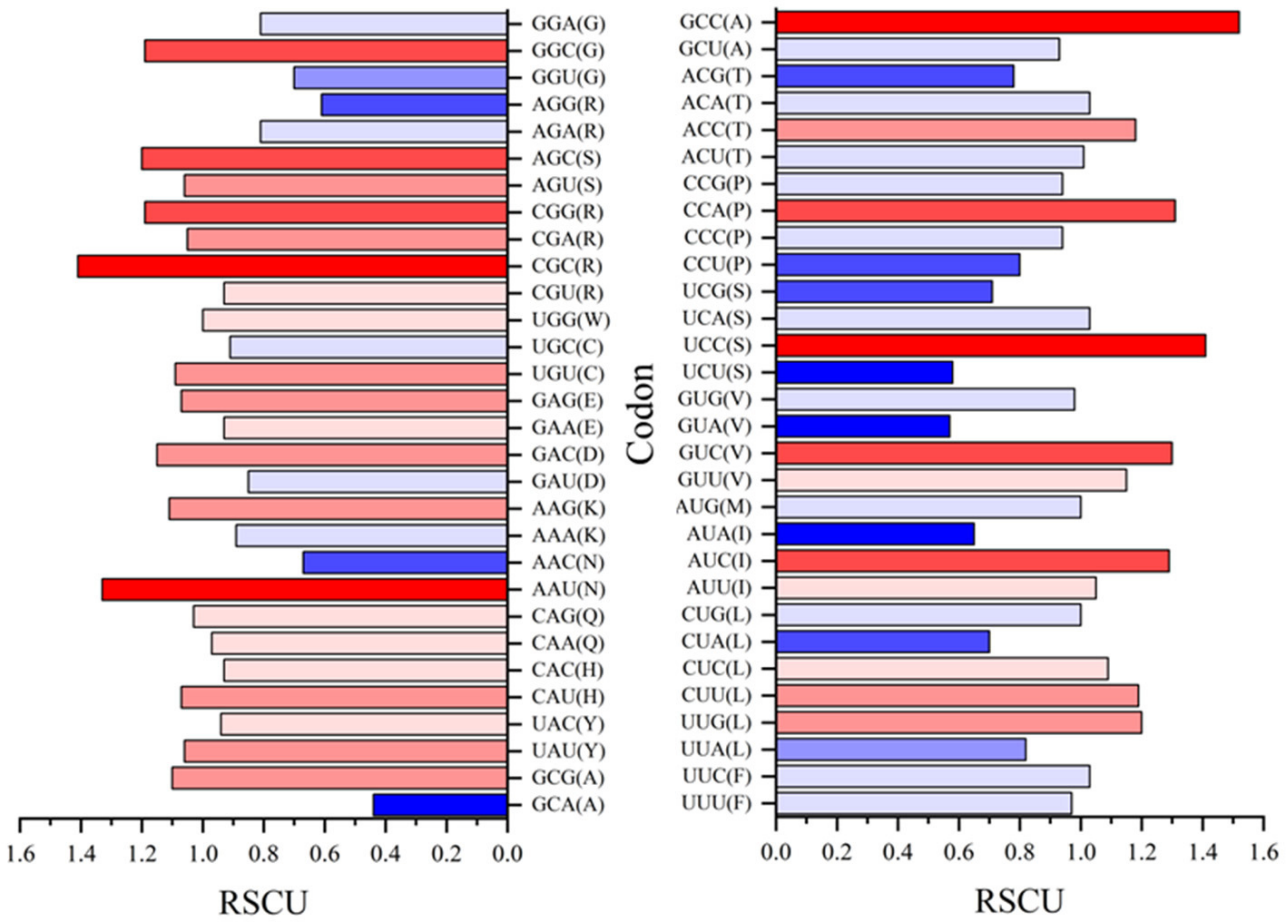
The relative synonymous codon usage (RSCU) values of 61 codons encoding 20 amino acids. This analysis was performed using Mega v11 on 36 *rep* sequences in GenBank and seven *rep* sequences of this study.

**Figure 11:**
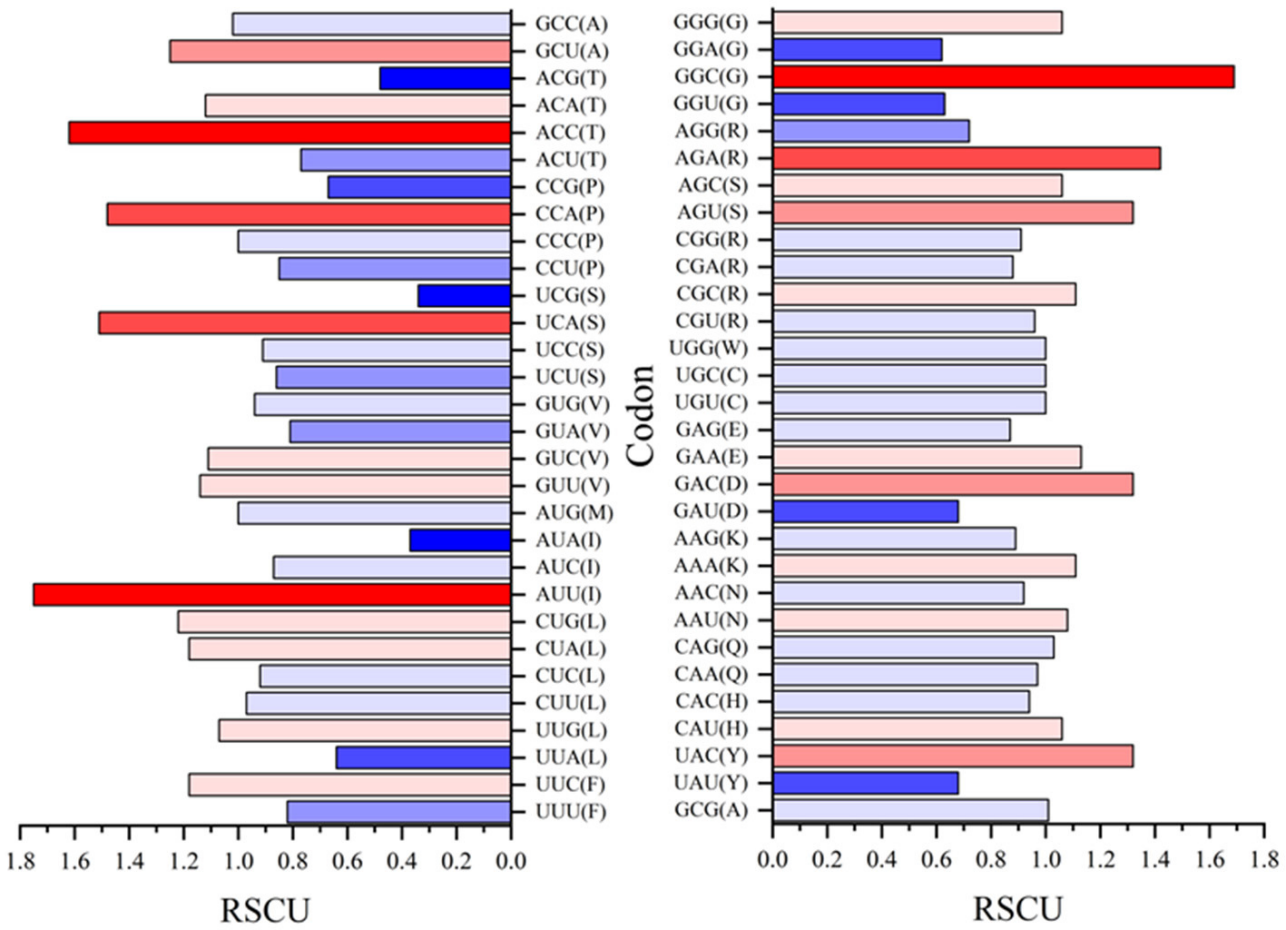
The relative synonymous codon usage (RSCU) values of 61 codons encoding 20 amino acids. This analysis was performed using Mega ver. 11 on 25 *cap* sequences in GenBank and 10 *cap* sequences of this study.

Relative synonymous codon usage (RSCU) values for both *rep* and *cap* are shown in Figures 10, 11, respectively. Figure 10 shows CGC(R), GGC(G), UUG(L), and GUU(L) to be the most abundant codon usage in *rep* sequences. The analysis described in this section is aligned with the EP values presented in the Figure 8 in which the codons associated with production of arginine (CGC) showed a 1.4 RSCU. The GCC codon associated with amino acid alanine had the highest RSCU value. Another interesting result related to codons associated with *rep* sequenced samples was the distribution pattern of codon usage. Figure 11 shows the RSCU calculated values for the *cap* sequenced sequence. As shown in this figure, GGC(G), AGA(R), CUG(L), and CUA(L) were the abundant codons within the *cap* sequence. When discussing comparison between the *rep* and *cap* on the codon RSCU values, it can be observed that the RSCU values of *rep* lie mostly in a range with less deviation, while the deviation within *cap* is broader, implying that a smaller group of codons are more frequently involved in production of amino acids within the *cap* sequence than the wider group of codons corresponding to the *rep* sequence.

### Variant analysis

To detect variants, two *rep* and six *cap* genomes suspected to be a new BFDV variant were selected, and Table 3 shows the suspected hosts and group information used for variant analysis. To accomplish this, two reference groups within each gene type were defined as: (1) GenBank-published BFDV sequences and non-suspected hosts sequences (sequences from this study with high similarity with GenBank-published sequences) and (2) suspected groups containing sequences from selected infected hosts. Table 3 shows the results from determining mean diversity distances and standard deviations. An increase in value above 0.05 indicates a new variant, signifying is a considerable amount of divergence between two groups. The mean diversity distances between the suspected group and the reference group for *rep* and *cap* were 0.07 and 0.15, respectively. A low standard deviation (0.01) also provided evidence that the mean diversity variation did not interact with the 94% threshold of new variants. Accordingly, the nominated BFDV variant of *rep* sequence had a similarity range of 92–94% with the reference group, while the suspected sequences of *cap* had a similarity range of 84–86%. Among all seventeen sequences, two *rep* and six *cap* BFDV-infected species can be considered to be new variants. Phylogenetic tree analysis, as well as determination of the mean diversity distance between groups exceeding 0.05 in both the *rep* and *cap* sequences, leads to the conclusion that the sequence associated with one infected budgerigar *rep* represents a distinct BFDV strain.

**Table 3:** Mean diversity distance between suspected species and reference groups including GenBank and non-suspected sequences.

**Sequence type**	**Group**	**Species**	**Mean diversity distance between groups ±Standard deviation**
*rep*	Suspected group	Lovebird	0.07 ± 0.01
		Budgerigar	
	Reference group	All considered *rep* sequence except two suspected species	
*cap*	Suspected group	African gray parrot	0.15 ± 0.01
		Cockatiel	
		Lovebirds	
		Conure	
		Budgerigar	
		Cockatoo	
	Reference group	All considered *cap* sequences except six suspected species	

Mega ver. 11 was used to compute the mean diversity distance among subpopulations based on 1,000 bootstrap values and P-distance method.

It is important to note that this study concentrated on 17 DNA sequences; additional DNA sequences are needed for a better understanding of BFDV genetic distribution in the psittacine bird population of Iran, as well as for identifying new strains of the virus. Also, in this study, the BFDV prevalence investigation was conducted on all psittacine birds regardless of their clinical symptoms. Further research should be conducted on the severity and clinical symptoms of BFDV infected birds in order to determine the percentage of infected birds that unhouse and conceal the symptoms of BFDV, since it is generally believed that some BFDV infected birds may not show the main clinical signs of the disease. Furthermore, the results of this study indicated that the role of exchange and trade markets contributed to BFDV spreading. Therefore, it is recommended to investigate this relationship more thoroughly by using time history phenotypic analysis of wild infected birds. This study examined feather samples only; therefore, an investigation on virus isolation through blood and tissue samples is recommended as well. Identifying the rare codon position in the BFDV, is a great leap toward advancing the way toward DNA vaccine development. Thus, the authors believe that further research is required in order to make a significant contribution to the prevention of BFDV within psittacine birds.

## Conclusion

The analysis described in this study suggests that the beak and feather disease virus (BFDV) is the foremost pathogen existing among more than 12 psittacine species in Iran. Phylogenetic analysis showed that BFDV sequences published in GenBank from Poland, Saudi Arabia, South Africa, Taiwan, and Thailand had the highest similarity with the BFDV sequences in the current study. Because of the large population size of some of those hosts, BFDV's transmission and mutation threats exist. According to the geographical analysis, psittacine birds inhabit a large area of the center and north of Iran, where there are national parks and forests. The evolution analysis led to a conclusion that the R, L and G are the three amino acids most frequently involved in the least-conserved substitution patterns of BFDV, ePerm. Conversely, M, Q, W are the amino acids that exhibited ultra-high conservation through the substitution patterns. The higher contribution of ePerm amino acids within sequence evolution is attributed to the substitution rate of R→ K and G→ S amino acid conversion. At the same time, the relative synonymous codon usage (RSCU) of *cap* and *rep* sequence analysis suggests that fewer codons [i.e., GGC(G)] of higher frequency is used in the c*ap* sequence while the greater number of codons with moderate frequency is observed in the *rep* sequence. The findings explain the wider distances observed through *cap* analysis compared to *rep* sequences. Finally, throughout the course of analysis of diversity between suspected hosts with respect to new BFDV variant and reference group, data analysis introduced a new variant of BFDV in *rep* and *cap* sequences of a budgerigar. Although six new suspected variants were identified, further study seeking stronger evidence is recommended.

## Data availability statement

The datasets presented in this study can be found in online repositories. The names of the repository/repositories and accession number(s) can be found below: https://www.ncbi.nlm.nih.gov/genbank/(OP039985-OP040001).

## Ethics statement

The animal study was reviewed and approved by the research committee of the Faculty of Veterinary Medicine, University of Tehran. Written informed consent was obtained from the owners for the participation of their animals in this study.

## Author contributions

SD, SP, and JR designed the study, analyzed the data, contributed to writing, critically reviewed the manuscript, and assisted in analysis of data. SD performed the study and wrote the first draft of the manuscript. All authors contributed to the article and approved the submitted version.

## Funding

This work was supported by the Research Council of the University of Tehran (Grant No. 7508007-6-42).

## Conflict of interest

The authors declare that the research was conducted in the absence of any commercial or financial relationships that could be construed as a potential conflict of interest.

## Publisher's note

All claims expressed in this article are solely those of the authors and do not necessarily represent those of their affiliated organizations, or those of the publisher, the editors and the reviewers. Any product that may be evaluated in this article, or claim that may be made by its manufacturer, is not guaranteed or endorsed by the publisher.
